# Gender disparities in historical context, workforce structure, and decision-making

**DOI:** 10.1007/s00066-026-02538-9

**Published:** 2026-05-27

**Authors:** Lisa Weissmann, Katharina Hintelmann, Stefanie Corradini

**Affiliations:** 1 Department of Radiation Oncology, Uniklinikum Erlangen, Friedrich-Alexander-Universität Erlangen-Nürnberg, Universitätsstr 27, 91054 Erlangen, Germany; 2 Translational Radiation Biology, Department of Radiation Oncology, Uniklinikum Erlangen, Friedrich-Alexander-Universität Erlangen-Nürnberg, Erlangen, Germany; 3 Comprehensive Cancer Center Erlangen-EMN, Erlangen, Germany; 4 Department of Radiotherapy and Radiation Oncology, University Medical Center Hamburg-Eppendorf, Martinistr. 52, 20246 Hamburg, Germany

Radiation oncology plays a crucial role in modern cancer therapy, as about 50% of cancer patients receive radiotherapy (RT) [1]. Ongoing technological advancements, improved treatment strategies and radiation techniques focus mostly on mechanical and anatomical precision. Furthermore, individualized treatment regimens in radiation oncology are designed to maximize tumor control while minimizing radiation exposure to surrounding normal tissues, thereby improving patient safety and reducing treatment-related toxicity [2]. However, a fundamental but often overlooked aspect of both research and treatment planning is the consideration of gender-specific responses in radiation oncology. This, however, could become increasingly more important, as the Organization for Economic Co-operation and Development (OECD) reported that from 2000 to 2022 age-standardized cancer-incidents showed an increase of 10% in women and 2% in men [3].

While sex-specific differences in the efficacy of pharmacological treatments, including chemotherapy, are well established, corresponding differences in response to ionizing radiation remain insufficiently characterized. Nevertheless, such differences are plausible and should be anticipated, particularly in light of documented sex-specific variations in radiation-induced cancer risk and DNA-mediated mechanisms influencing radiation response [4, 5]. These factors are likely to influence treatment outcomes and side effects, emphasizing their importance and should, thus, be considered for optimal patient outcomes.

While optimal clinical patient care, based on clinical and biological data, is the essential aspect of radiation oncology, gender effects are also visible in psychosocial and communication differences with patients and patient relatives [4, 6, 7]. Furthermore, structural, gender-based differences are present in the workforce itself [8–11], which is especially important, given the rising need of well-trained young professionals [11, 12].

## Gender disparities in historical context, workforce structure, and decision-making

A historical analysis assessed how women were actively involved in operating radiological equipment, developing imaging techniques, and contributing to radiation physics and therapeutic applications, following the discovery of X‑rays in 1895. Despite these contributions, women faced structural barriers including restricted access to university education, limited professional recognition, and discrimination within a male-dominated scientific environment. Several female pioneers contributed to key developments in the field, while also experiencing significant occupational risks, including radiation-induced malignancies [10]. Contemporary workforce analyses indicate that gender disparities persist within radiation oncology. Although the representation of female professionals has increased, they remain underrepresented in leadership and decision-making positions [8, 9, 13]:

Data from the Workgroup Women in Radiation Oncology (*Frauen in der Radioonkologie, *FiRO) from the German Society of Radiation Oncology (*Deutsche Gesellschaft für Radioonkologie*, DEGRO) show that female representation in the society has increased over time, reaching 45.1% of members in 2024. Women are well represented at early career stages, accounting for the majority of residents and specialists. While doctoral degrees are distributed relatively evenly between genders, representation decreases at higher career levels, with women comprising of 27% of leadership positions and 18.9% of full professorships [8]. This trend is not only visible in Germany, but also, for example, in North America [14].

This is also reflected in the participation in oncological guideline development: In general, participation of radiation oncology as a field is relatively high and radiation oncologists were present in 76.3% of the 93 identified oncological guidelines in the AWMF (*Arbeitsgemeinschaft der Wissenschaftlichen Medizinischen Fachgesellschaften e.* *V.*) registry, with particularly high involvement in S3 guidelines (92.5%). Nevertheless, women’s participation in these guidelines is lower than men’s. Among radiation oncology representatives, 255 mandate holders (out of 2795 mandate holders in total) were identified, with women accounting for 34.5% (28.3% among total mandate holders) and men for 65.5% of mandate holders [9]. Moreover, academic qualifications among all guideline participants showed a predominance of men among professors (81.3%), while women were more frequently represented among participants without academic titles [9], also reflecting the data of Besserer et al. [8].

Potential reasons for this observation are given in works from Trommer et al. [13] and Cairo et al. [15]: A survey of 218 young professionals in radiation oncology, biology, and medical physics examined employment conditions, workload, and career barriers, including unpaid care work [13]. One-third of participants reported care responsibilities. Female physicians and biologists with care responsibilities were more often in temporary positions, while male participants more frequently held permanent contracts. Work patterns differed between disciplines: Physicians often conducted research outside regular hours, whereas medical physicists and biologists did so within working hours. Participants with care responsibilities reported reduced research involvement. Workplace organization varied, with 37% lacking cover arrangements and 48% compensating missed work during evenings. Perceived support differed by gender and discipline, and reported risk factors included economic pressure, work–life balance, and compatibility of career and family [13]. A recent population-based analyses [15] showed gender-specific effects of parenthood on academic careers. While career trajectories are similar before parenthood, they diverge after the first child, with about one-third of women leaving academia alongside a reduced likelihood of attaining tenure, whereas no comparable effect is observed for men. The report concludes that these differences are not explained by career aspirations alone but by childcare responsibilities and working patterns. Women report greater caregiving involvement and reduced working hours after childbirth, with effects more pronounced in competitive environments and settings with fewer senior female role models [15].

Thus, gender differences exist not only in workforce composition, but extend into academic progression with potential influence over clinical decision-making, according to recent publications.

## Sex-specific differences in treatment outcomes and emerging biological differences

Recent clinical and translational analyses describe sex-specific differences in treatment response and outcomes in radiation oncology across multiple disease settings and therapeutic approaches. A retrospective analysis of 188 patients with anal squamous cell carcinoma treated with definitive radio(chemo)therapy demonstrated significant sex-specific differences in clinical outcomes. Female patients exhibited significantly improved overall survival, disease-free survival, and colostomy-free survival compared to male patients. In multivariate analysis, male sex, older age, and advanced tumor stage were identified as risk factors for poorer outcomes. Stage-dependent effects differed between sexes, with earlier tumor stages associated with improved outcomes in male patients, while no significant stage-dependent differences were observed in female patients [5].

These clinical observations are complemented by emerging data on sex-specific differences in a multicenter cohort study evaluating combined RT and immune checkpoint inhibition. Among 142 patients with metastatic solid tumors, abscopal response and tumor control occurred more frequently in female patients compared to males. While overall survival did not differ significantly between sexes, additional prognostic factors were identified, including the interval between immune checkpoint inhibition and RT and body mass index, which were associated with survival in both sexes. In contrast, elevated C‑reactive protein levels were associated with worse survival outcomes in male patients only [16]. Further preclinical and translational studies describe sex-specific differences in radiosensitivity, immune response, and toxicity, with females showing higher radiosensitivity in some contexts, but greater tolerance in organs such as lung and heart, influenced by hormonal, immune, genetic, and X chromosome-related mechanisms. This results in female survival advantages in some settings and inconsistent findings in others. Observed differences in toxicity include cardiac thresholds, neurocognitive effects, and hematologic toxicity, while epidemiological data indicate variation in radiation-associated cancer risk between sexes [17, 18].

Sex-specific differences may also extend to technical delivery parameters: A real-world study of 116 rectal cancer patients treated with volumetric-modulated arc therapy (VMAT) reported significant differences in setup errors between male and female patients. Correspondingly, required planning target volume (PTV) margins were smaller in males than in females, while bladder filling status was not associated with setup errors. These findings suggest that sex-related differences may also be relevant for setup accuracy and margin definition in pelvic RT [19].

Taken together, these studies describe sex-specific differences in outcomes, treatment tolerance, immune responses, and underlying biological mechanisms in radiation oncology [5, 16–18], with potential impact on treatment setup [19] suggesting that sex should not only be treated as a demographic, but also as a clinical variable.

## Sex-Specific differences in psychosocial aspects and patient experience

Beyond biological and clinical effects, gender-related differences also extend to the psychosocial domain, potentially influencing patients’ experience of RT and their interaction with supportive care [4, 6, 7]. A retrospective single-center study by Dinapoli et al. [4] examined gender-related differences in psychosocial outcomes among adult (*n* = 750) and pediatric (*n* = 145) cancer patients undergoing RT with psycho-oncological support from 2020–2024. Adults were assessed using the Distress Thermometer (DT) and Hospital Anxiety and Depression Scale (HADS), while pediatric patients received MapRT-based evaluation. Among adults, female patients reported significantly higher distress, anxiety, and depression than males (*p* < 0.001). While psychological outcomes varied by cancer type and disease-specific differences were observed, sex was a significant predictor in multivariable analysis. Utilization of psychological support also varied by diagnosis, with higher uptake in breast and gynecological cancers than prostate cancer. In pediatric patients, no sex differences in distress, anxiety, or depression were observed. Outcomes were similar across treatment intent groups, and prior psychopathologies showed no significant effect [4].

Taken together, gender-related differences in radiation oncology are present across multiple domains, such as workforce structure, clinical outcomes, biological response, and psychosocial factors, but are not yet systematically considered in research or clinical practice.

## Data Availability

Material used in this manuscript are available from the corresponding author upon reasonable request.
